# Supplementary material for: Views and experiences of maternal healthcare providers regarding influenza vaccine during pregnancy globally: A systematic review and qualitative evidence synthesis
Source: PLoS One. 2022 Feb 10;17(2):e0263234. doi: 10.1371/journal.pone.0263234 (PMC8830613; doi:10.1371/journal.pone.0263234)
Supplement: S3 Table — (DOCX) [file pone.0263234.s003.docx]

**S3 Table. Example of search strategy**

|  | **Medline (Ovid) search strategy** |
| --- | --- |
| 1 | (Maternity healthcare professionals).mp. [mp=title, abstract, original title, name of substance word, subject heading word, keyword heading word, protocol supplementary concept word, rare disease supplementary concept word, unique identifier, synonyms] |
| 2 | Maternity healthcare professionals.ti,ab. |
| 3 | (obstetrics & gynecology).mp. [mp=title, abstract, original title, name of substance word,subject heading word, keyword heading word, protocol supplementary concept word, rare disease supplementary concept word, unique identifier, synonyms] |
| 4 | Obstetric & gynecology, ti.ab. |
| 5 | (Antenatal care providers).mp. [mp=title, abstract, original title, name of substance word, subject heading word, keyword heading word, protocol supplementary concept word, rare disease supplementary concept word, unique identifier, synonyms] |
| 6 | Antenatal care providers, ti.ab. |
| 7 | (Midwives).mp. [mp=title, abstract, original title, name of substance word, subject heading word, keyword heading word, protocol supplementary concept word, rare disease supplementary concept word, unique identifier, synonyms] |
| 8 | Midwives, ti.ab. |
| 9 | 1 or 2 or 3 or 4 or 5 or 6 or 7 or 8 |
| 10 | exp influenza vaccine/ |
| 11 | exp maternal immunisation/ |
| 12 | (influenza vaccination).mp. [mp= title, abstract, original title, name of substance word, subject heading word, keyword heading word, protocol supplementary concept word, rare disease supplementary concept word, unique identifier, synonyms] |
| 13 | 10 or 11 or 12 |
| 14 | Views. mp. [mp= title, abstract, original title, name of substance word, subject heading word, keyword heading word, protocol supplementary concept word, rare disease supplementary concept word, unique identifier, synonyms] |
| 15 | Views, ti.ab |
| 16 | Knowledge.mp. [mp= title, abstract, original title, name of substance word, subject heading word, keyword heading word, protocol supplementary concept word, rare disease supplementary concept word, unique identifier, synonyms] |
| 17 | Knowledge, ti.ab. |
| 18 | Attitude.mp. [mp= title, abstract, original title, name of substance word, subject heading word, keyword heading word, protocol supplementary concept word, rare disease supplementary concept word, unique identifier, synonyms] |
| 19 | Attitude, ti.ab. |
| 20 | Practice.mp. [mp= title, abstract, original title, name of substance word, subject heading word, keyword heading word, protocol supplementary concept word, rare disease supplementary concept word, unique identifier, synonyms] |
| 21 | Practice, ti.ab. |
| 22 | Behaviour.mp. [mp= title, abstract, original title, name of substance word, subject heading word, keyword heading word, protocol supplementary concept word, rare disease supplementary concept word, unique identifier, synonyms] |
| 23 | Behaviour, ti.ab. |
| 24 | 14 or 15 or 16 or 17 or 18 or 19 or 20 or 21 or 22 or 23 |
| 25 | 9 and 13 and 24 |
| 26 | limit 25 to (English language and yr="2012 -Current") |
