# Supplementary material for: Views and experiences of maternal healthcare providers regarding influenza vaccine during pregnancy globally: A systematic review and qualitative evidence synthesis
Source: PLoS One. 2022 Feb 10;17(2):e0263234. doi: 10.1371/journal.pone.0263234 (PMC8830613; doi:10.1371/journal.pone.0263234)
Supplement: S4 Table — (DOCX) [file pone.0263234.s004.docx]

**S4 Table. Characteristics of included studies**

| **Paper and Country** | **Healthcare setting** | **Participants and sampling** | **Methodology** | **Analysis method** | **Aim** | **Findings** | **Recommendation** |
| --- | --- | --- | --- | --- | --- | --- | --- |
| **Bergenfeld et al., 2018**  **Kenya** | Not specified, public facilities | 111 Nurses and clinical officers.  Purposive sampling | Semi-structure interviews | Thematic analysis using hybrid (deductive and inductive) approach | Describe issues surrounding acceptance  and demand creation for maternal vaccines in Kenya from a provider  perspective | 1) trust relationship between HCP and pregnant women can enhance the uptake of influenza vaccine  2) Cultural, religious, and social factors influencing vaccine acceptance among patients; decision about vaccine intake can be controlled by many factors  3) Resources needed for improved vaccine delivery; time constraints and work overload may contribute to low recommendations for maternal vaccine  4) HCPs knowledge about maternal vaccines; continuing workshops about vaccination significance can improve vaccine delivery  5) Favourable attitudes toward maternal vaccines; attitude is related to knowledge and trust about the recommended vaccine  6) Access issues; long journey to healthcare facilities and cost are major barrier to vaccine delivery | 1.HCPs education about communications skills to promote vaccination  2.Decentralisation may help in publish regionally tailored resources for both patients and providers for better communication approach  3. Incorporating cultural and religious influencers to facilitate the introduction of new maternal vaccines. |
| **Maher et al., 2014**  **Sydney, Australia** | General practitioner practices | 17 General practitioners.  Purposive sampling | Semi-structured interviews | Thematic analysis | Investigate knowledge, attitude, beliefs and practices of GPs toward maternal influenza vaccine | 1)GPs’ risk perception of influenza during pregnancy, conflicts on risk perception toward influenza vaccine  2)GPs knowledge and perception toward maternal influenza vaccine, lack of knowledge about the importance of the vaccine may result in low coverage rate and  3)GPs approach to promote and provide maternal influenza vaccine, it is related to their vaccine risk perception and safety | Providing education and communications skills to GPs would enhance the recommendation and the delivery of maternal influenza vaccine |
| **Frew et al., 2018**  **Georgia and Colorado,USA** | Obstetricians & gynaecology practices | 24 Physicians, nurse practitioners, midwives, nurses, medical assistants and practice managers.  Purposive samoling | In-depth interviews | Thematic analysis using hybrid (deductive and inductive) approach | Identify clinic, provider, and staff-related attributes and facilitators to be utilized for a comprehensive vaccine intervention in ob-gyn clinical settings. | 1) strong provider “buy in” for maternal immunization; HCPs can enhance the uptake of influenza vaccine by using resources such as brochures and websites  2) the supporting role of clinical/interpersonal cues for vaccine promotion; by health education using posters  3) varying provider-patient communication approaches and its influence on maternal and pediatric uptake; HCPs recommendation for vaccine uptake is a major enhancer that is why the coverage rate is varying among different regions  4) A need for a designated office immunization champion; set specified personnel to vaccine promotion  5) reimbursement and practice implementation challenges; cost of the vaccine is a major barrier for its delivery  6) region differences in attitudes and values toward maternal immunization; Favourable HCPs attitude will result in high vaccine coverage rate | 1.Provide education intervention to improve vaccination delivery in obs&gyn setting  2. Provider guidance on how to recover costs from the implementation of vaccination programme in practice settings |
| **Li et al.,2018**  **China** | Tertiary hospitals | 18 Obstetricians.  Purposive sampling | In-depth interviews | Thematic analysis, inductive, constant comparative method | To understand HCPs’ perception and attitude toward maternal influenza vaccine | 1)Barriers to recommend maternal influenza vaccine including lack of awareness and knowledge about the importance of the vaccine, unfavourable attitude toward vaccinations and vaccine hesitance by pregnant women  2)Motivators for recommending maternal influenza vaccine including establishment of national policy, good knowledge about the vaccine significance, local evidence based, pro-vaccine social norms | 1)Prioritizing maternal influenza vaccine by national policy will enhance the delivery of the vaccine to pregnant women  2) Training on technical guidelines related to influenza vaccine will enhance vaccine recommendation for pregnant women |
| **Kaufman et al., 2019.**  **Melbourne and Perth, Australia** | Tertiary hospitals | 12 Midwives.  Snowballing samoling | Semi-structured interviews | Hybrid (Inductive and deductive) approach | Explore midwives’ attitudes, values and delivery of maternal vaccine to inform the design of a feasible and acceptable vaccine communication intervention package building on an evidence-based model utilized with US obstetricians. | 1) Who are midwives, describing their roles and values  2) How do midwives communicate about and/or deliver vaccines; vaccine recommendation and vaccine delivery perception  3) When and how much vaccine information do midwives provide; the time their spend to communicate with pregnant women about the significance of vaccine uptake  4) Where do midwives practice and communicate, the available resources and trainings  5) What vaccination resources are available or needed, suggested resources for future  6) Parents knowledge and attitudes;  7) Barriers and enablers to vaccination delivery and/or implementation of a vaccine promotion intervention. | 1. A need of multi-component intervention to optimize midwives’ vaccine discussions with expectant parents in the Australian context. |
| **Webb et al., 2014.**  **Adelaide, Australia** | Tertiary teaching hospital | 15 GPs, obstetricians, and midwives.  Purposive sampling | Semi-structured interviews | Thematic analysis | Explore the current practice of HCPs regarding maternal vaccine uptake and the interaction of knowledge, attitudes, beliefs, and practice. | 1) Barriers to implementing vaccine recommendations including undefined HCPs responsibilities toward vaccine recommendation, lack of vaccine uptake documentation and education level of pregnant women or/and HCP  2) Barriers to accessing immunizations including cost and unable to access due to lack of GP registration  3) Being part of a structured or systematic process; introduced as routine practice | 1)Introduce influenza vaccine into routine pregnancy care to eliminate logistical barriers and deliver the structures needed to ensure women are routinely offered these interventions.  2) Timing of vaccine delivery would help HCPs to support vaccination |
| **Wilson, 2019.**  **London,UK** | General practitioner practices | 10 GPs, midwives and practice nurses.  Purposive sampling | In-depth interviews | Thematic analysis hybrid approach | Understand access to and attitudes towards maternal vaccination among HCPs | 1) HCPs’ views towards maternal vaccination varies from accepting to considering the safety of the vaccine  2) Patient-healthcare professional relationships play and important role in improving the coverage rate of vaccine | 1)Ethnographic engagement in healthcare would allow wider understanding of vaccine hesitancy among HCPs. |
| **Fleming et al., 2018.**  **El_Salvador** | Not specified | 70 Community (leaders, community health personnel), public health managers and experts (national and international levels), policymakers, physicians.  Purposive sampling | Key informant interviews and semi-structured interviews | Thematic analysis | To share experiences from El-Salvador about maternal influenza vaccine, as it has high coverage rate | 1)MHCPs need education intervention to improve the delivery of maternal influenza vaccine  2)Prioritising maternal influenza vaccine in routine practice and campaigns help in improve the coverage rate  3)HCPs have direct influence on pregnant women decision related to uptake of vaccine  4)long distance, cost and violence are main barriers to access vaccine in unsafe regions of El-Salvador | Introduction of maternal immunisation into routine antenatal settings |
