# Supplementary material for: Views and experiences of maternal healthcare providers regarding influenza vaccine during pregnancy globally: A systematic review and qualitative evidence synthesis
Source: PLoS One. 2022 Feb 10;17(2):e0263234. doi: 10.1371/journal.pone.0263234 (PMC8830613; doi:10.1371/journal.pone.0263234)
Supplement: S5 Table — (DOCX) [file pone.0263234.s005.docx]

**S5 Table. CASP checklist for included studies.**

| **Paper** | **Was there a clear statement of the aims of the research?** | **Is a qualitative methodology appropriate?** | **Was the research design appropriate to address** | **Was the recruitment strategy appropriate to the aims of the research?** | **Was the data collected in a way that addressed the research issue?** | **Has the relationship between researcher and participants been adequately considered?** | **Have ethical issues been taken into consideration?** | **Was the data analysis sufficiently rigorous?** | **Is there a clear statement of findings?** | **How valuable is the research?** |
| --- | --- | --- | --- | --- | --- | --- | --- | --- | --- | --- |
| **Bergenfield et al., 2018** | Aim is clearly stated, importance, relevance and intended outcomes were articulated and justified. | Qualitative method is appropriate; the study is describing issues surrounding acceptance and demand creation for maternal vaccination in Kenya from provider perspective  by articulating why and how questions. | Research design was explicitly justified, author mentioned triangulation with pregnant women and key informant data to validate results | Choice of sampling was not justified; healthcare providers defined as physician, nurses, community health workers and clinical officers however, 111 HCPs including 97 nurses and 14 clinical officers no justification was mentioned about the inclusion of other HCPs | Semi-structured interviews data were collected in private rooms by two researchers team in English or Swahili. interviews were audio recorded, transcribed verbatim, translated when needed by the interview team. Data saturation was reached | Data collection was overseen by an anthropologist trained in qualitative methodology in partnership a Kenyan expert in maternal and child health; due to strike action some interviews were carried at participants own house which led to selection bias, over recruiting of HCPS from private sectors | Ethical approval was stated; written consent including interview participation, transfer of data for analysis and presentation of interview transcripts without identification. Ethical approval was obtained from Emory University | Data was analysed thematically; codes were created inductively and deductively NVivo software used to assess inter-coder reliability kappa >0.8 on 10 % of transcripts. Contradictory data finding was resolved, and themes were emerged accordingly | Findings are clearly described, providing evidence both for and against the uptake of maternal vaccine. they are presented in a structure reflecting the research questions; triangulation with pregnant women data from different study and key informant data were used to validate the results | The value of the study was considered, contribution to future interventions and suggestions for such as including a male family member in decision making intervention. Generalisability was not considered |
| **Maher et al., 2014** | Aim of the study was stated, its relevance and importance were articulated and justified | They seek to provide rich information on GPs’ perspective toward maternal influenza vaccine by asking why and how questions about knowledge, attitude and beliefs The research design was justified as the interviews as most appropriate to collect data to minimise recruitment burden and maximise recruitment rate | The research design was justified as the interviews as most appropriate to collect data to minimise recruitment burden and maximise recruitment rate | Purposive sampling was used to ensure diversity of participants as regard GP’s location (urban and rural), size and sex. Out of 666 GPs 44 GPs were participating, low participation due to workload for that recruitment was high to ensure data saturation | Semi-structured interview guide, using open-ended questions; interviews duration was 15 to 45 minutes, at GPs location supported by audio recorded. | The recruitment size was big to overcome low participation due to work overload. Nothing was mentioned as regard raising events during the study. The interview questions were guided with two non- participating GPs practitioners | Ethical issues were considered. The study was approved by the Sydney Local Health District Human Research Ethics Committee and consent was obtained from participants however, details about the consent were not provided | Four researchers conducted the analysis, conceptual framework was developed in the context to the aim of the study, NVivo software were used to develop codes using comparative approach. Each researcher develops a diagrammatic model for their codes then these models were synthesised. Contradictory data were not discussed | Findings were clearly described in the context of other published studies | The value of the study was considered, contribution to the future intervention and future research suggestions were discussed |
| **Frew et al., 2018** | Aim is clearly stated, importance, relevance and intended outcomes were articulated and justified | Qualitative method was appropriate as the authors seek to discover attitudes, practices, and experiences toward maternal vaccine in order to develop an intervention to enhance the uptake of the vaccine | The design was not explicitly justified | Choice of sampling was clearly justified. ob-gyn practices in Georgia and Colorado, to capture variety of provider experiences with range of racial/ethnic patient demographics | In-depth interviews at selected locations and times arranged with the clinic coordinator. data saturation reched; interviews ranged 30–40 minutes in duration. | The study discussed the social desirability bias however, researcher’s influence throughout the study including the formulation of research question, and data collection was not discussed. | Emory Institutional Review Board approval from Emory University was taken | The data collected from the interviews were transcribed, coded and analysed using NVivo software. these codes were refined. discussion about disagreements in coding and codebook refinement. | Findings were clearly described. Findings were discussed in the context of other published studies. They represented the findings with alignment to socioecological model. | The value of the study was considered as well as the contribution of future intervention. |
| **Li et al., 2018** | Aim of the study was clearly stated, importance, relevance and intended outcomes were articulated and justified | Qualitative method is appropriate to understand obstetricians’ perceptions and attitude related to the delivering of influenza vaccine during pregnancy | Research design was explicitly justified, to discover complex barriers such as cultural context and social norms by in-depth interview | Purposive sampling was explicitly justified, two obstetricians per hospital from five different sites in China to represent diversity of participant’s perception and attitude | In-depth interviews included six questions, conducted in hospital offices, lasted 25 to 35 minutes and were audio recorded. Data saturation was not discussed | Study potential bias was discussed, participants were females’ obstetricians recommended by chief obstetricians due to small number of male obstetricians in China another bias was small size participants; the interview was supervised by a staff member of the Center for Health Education which might have influenced on obstetrician feedback | Ethical issues were considered; oral consent was obtained from participants; ethical approval was obtained from the Center for Global Health of Centers for Disease Control and Prevention Office of the Associate Director of Science | Three researchers reviewed and coded using constant comparative method, themes emerged after three rounds of discussion. No discussion about the contradictory data | Findings were explicitly described in the context of other published studies | The value of the study was considered, contribution to future policymaking was discussed along with generalisability among China |
| **Kaufman et al., 2019** | Aim was clearly stated its relevance, importance and intended outcomes were articulated and justified | Qualitative method appropriate as they seek to explore midwives’ attitudes and values toward maternal vaccination, their perceived role in vaccine promotion and delivery, and barriers and enablers to implementation of a potential communication intervention | Research design was explicitly justified | Choice of sampling was justified. Justification of the selected site was to consider the impact of differences in healthcare delivery as dictated by state governments, and hospitals within states, who make independent decisions about funding, policy, and practice. | Data were collected by semi-structured interviews both telephone and face-to-face, based on scheduling availability and preference of the participant. Interviews lasted between 20 and 40 minutes. All interviews were audio-recorded and transcribed. The two interviewers used a single, open-ended question guide. Data saturation was mentioned | Researchers’ role in question formulation or data collection was not discussed, no discussion about implications of any changes in the research design. However potential bias was discussed due to small size sample and data saturation reaching fast resulting in homogeneous experience among participants which is not indicating generalisability | All participating midwives were asked to sign a consent however, no details were provided about the consent. All participants received a $25 card for their time. Ethics approval was obtained from authorities | Thematic analysis was performed on all interview transcripts were coding and themes were emerged using in NVivo software; they used template analysis to keep their analysis focused on the aim of the study. Further discussions were set in case of discrepancy in coding | Findings of the study were clearly described in the context of other published studies; respondent validation was not discussed; however, credibility of the data was mentioned | The value of the study was considered and contribution to future interventions and future suggestions. |
| **Webb et al., 2014** | Aim was clearly stated, and the relevance was articulated and justified | Qualitative method is appropriate to explore HCPs’ practice, knowledge, attitude, beliefs and practice toward maternal influenza vaccine | The research design was not explicitly justified | Recruitment choice was justified | Semi-structured interviews were conducted, digitally recorded and transcribed verbatim. The interviews applied open-ended question Data saturation was discussed | They aimed to develop ‘programmatic variation’ for that a purposive sampling was considered participants were stratified by occupation. They aimed to develop ‘programmatic variation’ for that a purposive sampling was considered participants were stratified by occupation | Research ethics approval was granted by the Children, Youth and Women’s Health Human Research Ethics Committee. However, no consent was mentioned in the study | One researcher coded all the data using NVivo software and a second researcher coded 3 interviews to ensure consistency in themes identified. Any differences between the 2 coding schemes were discussed and resolved with all researchers. Iterative approach was adopted, and themes emerged | Findings were clearly described and discussed in context of other published studies | Value of the study was considered, contribution for future intervention was discussed along with generalisability of the findings among other countries |
| **Wilson et al., 2019** | Aim was clearly stated, and its relevance was articulated and justified | Qualitative method is appropriate to gain a contextualised understanding of access to, and attitudes towards maternal vaccination among healthcare professionals in Hackney, London. | Research design was explicitly justified | Sampling choice was explicitly justified. Hackney was chosen as the study site as it has one of the lowest maternal vaccination coverage rates in England and diverse ethnicity and social standards. | In-depth interviews and a  videorecording at the GPs location. Interviews lasted for 20 minutes. However, data saturation was not mentioned | Potential bias was discussed as a small number of HCPs were interviewed due to work overload as well as all HCPs were women. The Hawthorne effect might occur during the consultation videorecording. No discussion about the implications of any changes during the study | No ethical considerations were discussed in the study | A thematic analysis was conducted to identify, analyse, and emerge themes, by ‘contextualist’ method; interview transcripts were uploaded into NVivo11; coding framework was formulated both deductively, and inductively. Contradictory data was not discussed | Findings were discussed in relation to other published studies | Value of the study was discussed. No discussion was made about generalisability and implications for future interventions |
| **Fleming et al., 2018** | The research aim was clearly stated, importance and relevance were articulated and justified | Qualitative method is appropriate; the study seeks to articulate answers to why and how questions about what might motivate HCPs to deliver influenza vaccine | Study design was not explicitly described | They conducted the study in two municipalities in each of El Salvador’s three regions. | interviews were conducted in Spanish and audio recorded; recordings were transcribed and translated into English data saturation were mentioned | There was not any discussion about the researcher role on data collection or analysis however, they mentioned potential selection bias due to sample recruitment in areas considered safe. | Ethical approval stated | A codebook was developed; NVivo software was used to conduct a thematic analysis of the data. However, no discussions about contradictory findings | Findings were clearly described; finding are considered is light of practice and policy; the implications were discussed in relation to other developing countries. | Author considered the value of the study and the contribution of future interventions. They discussed generalisability of the findings among LMICs |
