# Supplementary material for: Views and experiences of maternal healthcare providers regarding influenza vaccine during pregnancy globally: A systematic review and qualitative evidence synthesis
Source: PLoS One. 2022 Feb 10;17(2):e0263234. doi: 10.1371/journal.pone.0263234 (PMC8830613; doi:10.1371/journal.pone.0263234)
Supplement: Attachment — Submitted filename: Response to editors and reviewers 15 Aug 21.docx [file pone.0263234.s006.docx]

**Editors’ Comment**

| **Comment** | **Response** |
| --- | --- |
| 1. Please ensure that your manuscript meets PLOS ONE's style requirements | This has now been amended |
| 2. Thank you for providing your flowchart as figure S1. Please make this figure 1 in your main manuscript. | This now has been added |
| 3. Please confirm that you have included all items recommended in the PRISMA checklist including the full electronic Boolean search strategy used to identify studies with all search terms and limits for at least one database. Please attach this as supplementary file | This now has been amended and an example of search strategy has been added as supplementary file |
| 4. Thank you for stating the following in the Acknowledgments Section of your manuscript:   "KJ is supported by the National Institute for Health Research (NIHR) Applied Research Centre (ARC) West Midlands. The views expressed are those of the author(s) and not necessarily those of the NIHR or the Department of Health and Social Care."  We note that you have provided funding information that is not currently declared in your Funding Statement. However, funding information should not appear in the Acknowledgments section or other areas of your manuscript. We will only publish funding information present in the Funding Statement section of the online submission form.  Please remove any funding-related text from the manuscript and let us know how you would like to update your Funding Statement. Currently, your Funding Statement reads as follows:  "The author(s) received no specific funding for this work." | We would like to update the funding statement to read:  “The author(s) received no specific funding for this work. KJ is supported by the National Institute for Health Research (NIHR) Applied Research Centre (ARC) West Midlands.” |

*Reviewer One Comments*

| **Comment** | **Response** |
| --- | --- |
| 1. I would suggest adding the contributing studies, along with their quality and location, to Table 3. | The references to the contributing studies have been added to table 3. We do not think that it is necessary to add quality and location to table 3 as this is reported in table 2 and additional details will reduce the clarity of the message. |
| 2. I don't fully understand why this paper assessed the quality of each contributing study with the CASP but then does not utilize those assessments to eliminate, weight, or contextualize the interpretations of the findings. Instead, the methods include an assessment of data richness, but the assessment of this is not explained. | Critical appraisal within qualitative systematic reviews is debated within the review methods literature. Few qualitative systematic reviews use a quality appraisal to formally weight studies. We have highlighted in the methods the lack of consensus about the assessment of study quality in the synthesis of qualitative research [42]. Conceptual richness is distinct from methodological reporting quality. Conceptual richness relates to the relevance of each paper to the review objectives and how much rich data each paper contributed to the synthesis. A study can be poorly reported but still contribute rich data to the synthesis and vice versa.  Overall, the reporting quality of the studies was high, this is presented in a supplementary table. We have added additional text to the discussion highlighting the quality of the studies increasing the confidence in the findings.  *“Overall, the quality of the included studies was high, though there is a lack of consensus about the assessment of study quality in the synthesis of qualitative research [42].”* |
| 3. The % of countries which recommend influenza vaccine for pregnant women and if possible, the extent to which national guidance is available, should be included. Further, significant years (introduction of vaccine, change in guidelines, etc.) should be mentioned, and the years of the search strategy should be included. | We have added the date that WHO recommended flu vaccination in pregnancy (2012). We have also added vaccine coverage rates where these data are available/accessible It’s challenging to find this information. (See page 4)  The dates of the search (2012 to March 20220) were included in the “Search Strategy” section of the Methods. |
| 4. In the abstract, there is information in the conclusion not included in the results; new findings should not be presented here. | This now has been amended |
| 5. "decline" would be a better word than "rejection" | Thank you, we have made that change |
| 6. a review of effective interventions would be a different review; if the point here is to ask what MCHPs would recommend, please state that clearly. | We have amended the aim to clarify that it includes identifying interventions that MHCPs consider feasible to implement.  *“The aim of the study was to systematically review and thematically synthesise qualitative evidence that explores the views and the experiences of MHCPs involved in the provision of the maternal influenza vaccine worldwide. In addition, we looked for interventions that MHCPs consider feasible to implement to increase vaccination uptake”.* |
| 7. Please be careful with the framing as these are MCHPs often reporting on what they THINK is influencing women's uptake of the vaccine. In other research, we have seen that health care workers and patients have different understandings about reasons for patient behaviour. | Thank you, we have checked through the manuscript to ensure that we report that the views are from MCHPs, not directly from pregnant women. |
| 8. What terms are used in the search? Can the search strategy be appended? | The key search terms are included within the search strategy section of the methods. We have now added an example search strategy for one database as a supplementary file. |
| 9. It's a significant limitation to restrict to English-only. How many potential papers were screened out because of language? Please add further explanation in the limitations about the possible biases introduced because of this. | The search strategy was restricted to English language only, so we are unable to estimate this. We did undertake a search in Medline to see whether we had missed any non-English studies, but found none. We acknowledge this limitation in the discussion section. |
| 10. Why are community health workers included in the results, if they are not included in the inclusion criteria? Please clarify. | Community health workers were included under the group “health workers and public health personnel working in maternity healthcare settings”. We have now added “e.g., community health workers” to clarify this. |
| 11. Given how small the sample size is, the findings are far too general. For example, "MHCPs held one on one discussions" is only cited by one study, so cannot be generalized to every practice around the world. | Communication between MHCP and pregnant women have been mentioned in three studies and we have demonstrated different approaches which are: one-to-one discussion and using passive language [45, 48, 50]. |
| 12. The headings have reference to the MHCP and women's "characteristics" but I don't see them in the findings (other than the power dynamic between patient and provider, which seems more of a perception and less about the characteristics of the MHCP him/herself) | To avoid confusion, we have amended these, so the analytical themes are now: “MHCPs perceived views of pregnant women’s health literacy and beliefs” and “MHCPs knowledge and attitudes”. |
| 13. “Social and cultural norms" is quite a broad category - as it covers religion, intrahousehold power, and gendered dynamics. Consider separating these out. | We have now amended these, so the analytical theme is now: “social, cultural and environmental influences” and the descriptive themes are now: “religion, family influences, media influence and access issues” |
| 14. Consider adding identifiers to the quotes - what cadre of health care worker is speaking? From what country was the contributing study? | We have added the country. We have also added whether it was a primary quote (italics) or author interpretation (normal font). It is not possible to add the cadre of staff as not all primary quotes were identified in paper and some quotes are author interpretations. |
| 15. It seems that the prohibition on ANC might be one of the most important barriers to vaccination uptake rather than specifics around the vaccine itself. This gets lost in the current framing under social norms, but would certainly require a different set of interventions or messages | Please see our response to point 13. We have now amended these, so the analytical theme is now: “social, cultural and environmental influences” and descriptive themes are now: “religion, family influences, media influence and access issues”. |
| 16. "Media" is pretty broad - what types of media? (tv, internet, radio, social media, etc.) | We are unable to add additional detail and this is not mentioned/ specified in the study |
| 17. gang groups and violence around the clinic seems very specific to one site, do you have evidence that this is more widespread? (see above my comment about CERQual, as this method would downgrade a finding such as this) | Please see our response to point 2. We agree that this is an issue limited to one study, but it fits within the wider issues relating the access issues. |
| 18. In the discussion, while more confident providers may lead to higher uptake, there was a concerning quote in included in the paper about how patients may not feel empowered to decline something recommended; I would include a caveat here about the importance of informed consent. | We agree with the reviewer, now we have added this:  “It is however, important that MHCPs support informed consent for influenza vaccination which will provide a communication approach between MHCPs and pregnant women to discuss influenza vaccination” |
| 19. Workload and time constraints feature prominently in the abstract and discussion, but did not come out strongly in the findings as presented (other than deprioritizing this vaccine against others, but there were other confounders) | We have added additional results and quotes to support this:  “Workload and time limit were factors reported by MHCPs as limiting opportunities for influenza vaccination discussion with pregnant women [22, 50]:  *“Maybe when a health care provider is in a hurry or is being overworked, you may find a long queue at the ANC waiting for vaccination. The nurse there may not have time to discuss much with every client about the vaccines. Sometimes they issue orders for the mothers to queue and get vaccinated. These are situations which may happen when there are several mothers at the clinic. This can cripple vaccine uptake since there is no time for explanations”* [Kenya, 22]  *“The doctors are very busy and so for example in the private clinic we run at capacity so we’re turning women away, so we basically have to apply um err almost triage principles to how we run our consultations but we do have a um midwife there with us who is our personal sort of assistant if you like. So things like breastfeeding and err analgesia in labor and vaccinations although I don’t know if they mention vaccinations I’ll be honest. We tend to delegate to them. The longer we make the consultations basically the less patients we can see.”* [Australia, 50] |
| 20. Vaccine champions are mentioned but not explained | This has now been explained:  *The need for a vaccination champion who is managing and creating enthusiasm for vaccination campaigns as well as encourages better communication about vaccination between HCPs,* |
| 21. I don't understand the phrase "naturally present" with regard to patient-provider relationships; why would this be inherent? | We have amended this term to make the meaning clearer. We now say: “usually present” |
| 22. Table 3 is very unclear: are these the views of MCHPs about women's views? | Table 3 presents the key themes which summarise MHCPs’ views about influenza vaccination in pregnancy and what they think are the barriers to women being vaccinated. We have clarified this in the overall aim and re-worded the table caption which now reads:  “Core analytical and descriptive themes for MHCPs’ experiences and views relating to the provision of influenza vaccination for pregnant women” |
| 23. Please add line numbers so reviewers can more easily point out suggestions | Line numbers have been added |

*Reviewer Two Comments*

| **Comment** | **Response** |
| --- | --- |
| **Introduction** | |
| 24. See this recent study Dawood et al, Incidence of influenza during pregnancy and association with pregnancy and perinatal outcomes in three middle-income countries: a multisite prospective longitudinal cohort study - The Lancet Infectious Diseases for additional (though contradictory) data regarding risk of antenatal influenza and birth outcomes. | Thank you – we have added detail about this study. |
| 25. You can condense your first two sentences, as your readers know pregnant women and their fetuses are at high risk from influenza | Thank you – we have made this change |
| 26. Would make two sentences from Deberra et al study—one about effects of vaccine in pregnant women, one about protection in infants | Thank you – we have made this change |
| 27. Is there a published statistic for uptake of influenza vaccine in pregnancy globally? Looks like about 50% in United States (Low Rates of Vaccination During Pregnancy Leave Moms, Babies Unprotected ([cdc.gov](http://cdc.gov/))) | We have added this statistic for several countries but there does not appear to be a global statistic readily accessible  *“The two recommended vaccines during pregnancy are influenza vaccine and Tdap (Tetanus, diphtheria, and pertussis) vaccine. However,* *in 2019–20 in the United States, 61.2% of pregnant women received influenza vaccine, 56.6% received Tdap, and 40.3% received both vaccines [64]. A 2020 expert commentary review reported an 8.7% median influenza vaccine coverage rate in pregnant women in the European region [63], whereas in the US during the 2017–2018 influenza season, they reported a 49.1% influenza vaccine uptake before or during pregnancy. High rates have been* *achieved, with 95.7% uptake in Brazil in 2010 as a result of a government vaccination campaign [63].”* |
| 28. Brings up interesting point—there are (at least) two issues at play here: 1) How often are pregnant women being offered the influenza vaccine by their MHCP? 2) How often are women receiving the vaccine? Your paper is primarily concerned with the former, but this is never explicitly delineated. | We agree with the reviewer that there are potentially separate issues, although uptake/receipt of the vaccine is dependent on it being offered. We have drawn out this distinction in the introduction:  *“Questions such as how often pregnant women are being offered the influenza vaccine or how often pregnant women are receiving the influenza vaccine need to be addressed.”* |
| 29. When you say “experiences” for MCHPs (2nd sentence, 3 paragraph) do you mean MCHPs’ past experience recommending influenza vaccine? Knowledge, beliefs, and time constraints are fairly self-explanatory concepts, but “experiences” remains unclear to me throughout the paper. | We use the term ‘experiences’ within the paper to include MHCPs’ experiences with pregnant women in relation to discussing and delivering maternal influenza vaccine. |
| 30. Your fourth paragraph is strong and makes a good argument for the necessity of the paper. | Thank you |
| **Methods** | |
| 31. Well delineated inclusion and exclusion criteria, though would be interesting to comment on difference between MCHPs’ views of Tdap vs influenza vaccines (structural constraints identified as barrier to Tdap vaccination as well) somewhere in discussion. | The latest CDC statistics about Influenza and Tdap coverage rate has now been added to the introduction (lines 88 to 91). Whilst we agree that the contrast between the barriers for delivering Tdap and influenza in pregnancy is interesting, we feel that it would distract from the clarity of the narrative in relation to influenza vaccination. |
| **Result** | |
| 32. Table 2 informative | Thank you |
| 33. Quality assessment is useful. Seems like Bergenfeld et al was problematic from recruitment strategy perspective—may need to justify its inclusion (especially since it is heavily referenced in the introduction section) | Please see response to point 2 above. We didn’t specify any eligibility criteria in relation to quality of studies, and Bergenfield otherwise had high quality reporting, so we believe it was correct to include the study. |
| 34. Table 3 is clear delineation of themes | Thank you |
| 35. Figure 1. MHCP “characteristics” not clear—do you mean knowledge, attitudes, and beliefs? | To avoid confusion, we have amended these, so the analytical theme is now “MHCPs’ knowledge and attitudes”. See response to point 13 above. |
| 36. More generally, it seems like there is more to draw out of the “knowledge” and MHCPs. The “Characteristics” and the first part of “Local Healthcare system” sections seem like they would be better combined under “MHCP knowledge and education about influenza vaccine”, especially as this emerges as a target for intervention in your discussion. | We carefully considered the themes. However, as with all qualitative research and thematic-syntheses, different research teams might draw out slightly different analytical and sub-themes. |
| 37. Then the rest of “local healthcare system” is systems factors (checklist, electronic medical record-based interventions, place for documentation) which seems like its own theme. | Thank you for the thoughtful suggestion. We agree that training and education could be considered to fit within ‘local health care system’ or ‘MCHP’s knowledge and attitudes’. We would like to keep the training and education within the local healthcare system as the delivery of this is not something that should be the responsibility of an individual MHCP, but should be considered for the whole maternity health care system as a part of organised updates and ongoing training and development. |
| 38. The record keeping/vaccination history piece of “national policy and practice” is an excellent point, and perhaps should be highlighted in your discussion section as well | Thank you – we have now included this point within the discussion: |
| **Discussion** | |
| 39. Is there way to explicitly highlight MCHP generated strategies (visiting religious services in Kenya, creating EHR vaccination prompts, dedicated vaccine teams, education hub) to improve influenza vaccination? | This has now been added:  *“Moreover, MHCPs discussed strategies such as visiting religious services in Kenya”* |
| 40. In paragraph 4 of this section, last sentence should become two. This paragraph seems to be the crux of your argument—that MCHPs are uniquely positioned to affect uptake of influenza vaccine. | We have split the sentence with a semi-colon to clarify the meaning. We agree that this is an important finding. |
| 41. For paragraph 6, you don’t really bring this theme out in your thematic analysis in the results section. | Please see response to point 19 above. |
| 42. For paragraph 7, the vaccination champion idea also is not adequate introduced in the results section | Please see response to point 20 above. |
| 43. In paragraph 8, are you suggesting focusing resources on midwives especially? Would make this clear if this is the case. | The paragraph does suggest that midwives would be well placed for this role. |
| 44. In paragraph 9, last sentence is a sentence fragment—would revised | This sentence has been rephrased to improve its clarity. |
| **Conclusion** | |
| 45. The last sentence is a run-on and does not make sense. | The sentence has been re-written:  *Evidence-informed interventions are needed to improve influenza vaccination rates by addressing MHCPs hesitations as well as developing or improving local and national guidelines.* |
