# Supplementary material for: Views and experiences of maternal healthcare providers regarding influenza vaccine during pregnancy globally: A systematic review and qualitative evidence synthesis
Source: PLoS One. 2022 Feb 10;17(2):e0263234. doi: 10.1371/journal.pone.0263234 (PMC8830613; doi:10.1371/journal.pone.0263234)
Supplement: Attachment — Submitted filename: PLOS response 8 December 2021 KJ .docx [file pone.0263234.s007.docx]

Thank you for the opportunity to make changes to strengthen this manuscript. We thank the reviewer for their helpful and detailed comments which we have addressed below. Line numbers refer to the clean untracked version.

*Reviewer’s global comments*

|  | **Reviewer comment** | **Author response** |
| --- | --- | --- |
| 1 | Most of section 4.5.1 relates to barriers to ANC in general, not vaccines in particular | The included studies were only concerned about vaccine during pregnancy including influenza, so the statement reflects facts about influenza vaccine and other pregnancy vaccines as well. However, we have amended a sentence at the start of section 4.5.1 (line 235-238) to make this clearer that lack of access to care influences the ability to access the vaccine:  “Social and cultural norms, including religion, family involvement, media influence and access issues, were perceived by MHCPs to influence a pregnant women’s decision about influenza vaccination in pregnancy or access to antenatal care and thus the opportunity to be offered influenza vaccination.” |
| 2 | Would benefit from more consistent language throughout. The paper focuses on the perspectives of MHCP’s on the influenza vaccine (rather than knowledge, attitude, and beliefs). Among themes emerging from the qualitative studies reviewed are “social, cultural, and environmental influences,” “MHCP’s knowledge and belief” about the vaccines. This extends to the title—might try “Perceptions of Maternal Health Care Providers on …” | We have also tried to be consistent with language using views and experiences, rather than knowledge, attitudes and beliefs.  This is now consistent with the title: “**Views and experiences of maternal healthcare providers on influenza vaccine during pregnancy: a systematic review and qualitative evidence synthesis”** |
| 3 | The addition of country and study to the included quotes is very helpful. | Thank you |
| 4 | Still don’t see much critical evaluation of studies included in your discussion section—for example, issues around the heterogeneity of providers included makes the discussion of who is responsible for advocating for influenza vaccine confusing (MD/DO’s believing it is a nurse role) | The heterogeneity of MHCPs is now addressed in several places within the discussion (see responses to points 16, 18 and 28). We have added a sentence near the start of the discussion that raises the issues of boundary issues between different MHCPs (line 426-428):  “The range of different MHCPs who could be involved in promoting and delivering influenza vaccination can lead to boundary issues as to whose responsibility it is.” |
| 5 | Would benefit from more classification of findings in discussion: on barriers (workload, time constraints, MHCP’s perceptions of women’s concerns about flu vaccine, lack of knowledge on the part of MHCP re: flu vaccine), facilitators (good rapport, national guidelines, education/training) and suggestions for further intervention (vaccine champions, EMR prompts, systems level informatics regarding who is due for flu shot, free vaccines) | The discussion has been re-ordered so that a discussion of the barriers, facilitators and suggestions for interventions to increase vaccine uptake are explicitly covered within individual headings in the context of the wider literature. |
| 6 | This is a very timely study given the applicability to COVID vaccines in pregnancy—would probably be more explicit about this when it comes to significance—understanding the perspective of MHCP’s on influenza vaccine may shape strategy for educating them about COVID vaccine in pregnancy. | In the discussion (line 435-437) we have added:  “Our findings may have insights for the provision of vaccination against COVID-19 during pregnancy such as knowledge and beliefs perceived by the MHCPs toward the delivery of COVID-19 vaccination.” |

**Reviewer’s line by line comments**

|  | **Reviewer comment** | **Authors response** |
| --- | --- | --- |
| 7 | 26-28 Still unclear: “experience, knowledge, beliefs” of the MHCP’s themselves? Still vague—would reword for clarity, especially in abstract. This is broken down clearly in your thematic analysis so why not say “knowledge and attitude” and “social, cultural, and environmental influences.” | This now has been amended as (line 27-29):  However, factors such as MHCPs’ views and knowledge about the vaccine, and time constraints due to workload may influence MHCPs’ practices and opinions about women receiving the influenza vaccine during pregnancy. |
| 8 | 41-42 Same—are you saying that their commitment to provision of flu vaccine was challenged by workload and time constraints? By social, cultural, and environmental influences? By women’s perceived concerns? Perhaps cast these as facilitators: trusting relationships, good communication, knowledge about the vaccine leading to confidence in recommending vaccine, EMR prompts, and presence of national guidelines. And then the barriers: workload, time constraints, MHCP’s perception of pregnant women’s concerns, and social/cultural/environmental influences. | This now has been amended as (line 42-48):  “…facilitated by trusting relationships, good communication, knowledge about the vaccine leading to confidence in recommending vaccine, EMR prompts, and presence of national guidelines. However, workload, time constraints, MHCP’s perception of pregnant women’s concerns, and social/cultural/environmental influences could prevent the likelihood of delivery of influenza vaccine.” |
| 9 | 71 Don’t need “however” | This now has been amended |
| 10 | 83-86 should read: Evidence showed the vaccine offered immunity | This now has been amended |
| 11 | 90 I think you mean: In 2019-2020 in the US, 61.2% of pregnant women received influenza vaccine, 56.6% received Tdap, and 40.3 received both | This now has been amended |
| 12 | 89-96 These statistics seem contradictory—may need a sentence up front suggesting “….heterogeneity globally—from less than 10% coverage in Europe, 50-60% in US, and >95% in Brazil.” | This now has been amended as: (line 91-94)  “In 2019–20 the United States (US) reported that 61.2% of pregnant women received influenza vaccine, 56.6% received Tdap, and 40.3% received both vaccines [19]. A 2020 expert commentary review reported heterogeneity of coverage globally, from less than 10% coverage in Europe, to 50-60% in US, and >95% in Brazil [20].” |
| 13 | 98-101 likely a typo here affecting clarity: I think you are saying “MHCPs knowledge of and beliefs about the influenza vaccine, and their experiences supporting pregnant women, as well as their workload and time constraints, affect their practices in advocating for influenza vaccine during pregnancy. “ | This now has been amended |
| 14 | 109-110 These are fundamentally different questions, and your study seems to focus on the factors affecting the first of these. It’s an interesting and important point—maybe in focusing on pregnant women’s perspectives, we’re actually missing one of the main problems, which is that they are not being offered (or encouraged to take) the influenza vaccine. It follows then that is essential to understand the perspective of those offering/recommending the vaccine. | We have tried to clarify the focus of the review in relation to the questions (line 108-110):  “Questions such as how often pregnant women are being offered the influenza vaccine or how often pregnant women are receiving the influenza vaccine need to be addressed. This review largely focusses on the offer of the vaccine. The aim of the study was to systematically review and….” |
| 15 | 178 need a period at the end of this sentence. | Added, thank you |
| 16 | 198 will need to comment on the heterogeneity of this group in your discussion section | We have added a sentence to the discussion section (line 517-518):  “In addition, the MHCPs were a heterogeneous group, widening the transferability and providing a range of views and experiences.” |
| 17 | 235 4.5.1 None of these particular to vaccine uptake, they all seem more generally related to provision of ante-natal care. (257-260 reference is the exception here). This is obviously not prohibitive in including this section, but would just acknowledge that the barriers here are largely barriers to ANC itself | We have amended a sentence at the start of section 4.5.1 to make this clearer that lack of access to care influences the ability to access the vaccine (line 235-238):  “Social and cultural norms, including religion, family involvement, media influence and access issues, were perceived by MHCPs to influence a pregnant women’s decision about influenza vaccination in pregnancy or access to antenatal care and thus the opportunity to be offered influenza vaccination.”  We have also highlighted this relationship within the discussion section (limitations – lines 522-).  “Also, there may be findings that relate to a particular context or setting, in particular settings where access to antenatal care was the underlying cause of lack of access to influenza vaccination.” |
| 18 | 374 this is where the heterogeneity of your population is problematic—some of your MHCP are nurses | Please see earlier response about heterogeneity. We have also added it as a limitation within the discussion section (lines 528-531):  “Also, there may be findings that relate to a particular context or setting, in particular settings where access to antenatal care was the underlying cause of lack of access to influenza vaccination, or where professional group’s roles and responsibilities varied between settings” |
| 19 | 386 need semi colon between studies; it | This now has been amended |
| 20 | 386 they is unclear. Would say “vaccine champions” may increase | This now has been amended |
| 21 | 395: setting a national policy and guidelines, centralizing vaccine information, and providing influence vaccine for free would enhance… | This now has been amended |
| 22 | 430-433 these need separate sentences—they are all very different ideas. | We have split this into two sentences to address knowledge and uptake and delivery as separate issues (lines 430-435):  “Moreover, MHCPs discussed strategies to increase knowledge and vaccine uptake, such as visiting religious services in Kenya, and creating educational hubs for pregnant women. Suggestions to increase delivery of the vaccine included creating electronic vaccination prompts and a dedicated vaccine team.” |
| 23 | 433 take out “likely” | This now has been amended |
| 24 | 446 Would re-word for clarity | This now has been amended to (line 477-479):  “Taking informed consent for influenza vaccination from pregnant women might provide a communication opportunity between MHCPs and pregnant women to discuss the vaccination.” |
| 25 | 452 should read “for existing and new vaccines” | This now has been amended |
| 26 | 467-470 run on sentence | This is now has been amended as (line 481-484):  “Evidence based and effective training and education is a necessity for MHCPs to be aware of the importance of vaccinations as well as enabling them to discuss any hesitations that they may have, in order to cope with the expectations of the system and the concerns of pregnant women.” |
| 27 | 471 don’t need “however” | done |
| 28 | 481 maybe discussion of heterogeneity of providers included in this study goes here, and then highlight role of midwives | Thank you for the suggestion. It now reads (line 489-491):  “The range of MHCPs in the studies in this review were heterogeneous. However, as midwives spend more time with pregnant women than other MHCPs, they are ideally placed to be more engaged with vaccination promotion campaigns and programmes.” |
| 29 | 484 take out comma after pregnancy | done |
| 30 | 491 YES! Lots of overlap. This helps add validity to your findings (or WHO’s model…) Figure 2 I like this model | Thank you |
