# Supplementary material for: Views and experiences of maternal healthcare providers regarding influenza vaccine during pregnancy globally: A systematic review and qualitative evidence synthesis
Source: PLoS One. 2022 Feb 10;17(2):e0263234. doi: 10.1371/journal.pone.0263234 (PMC8830613; doi:10.1371/journal.pone.0263234)
Supplement: Attachment — Submitted filename: Respnses 11Jan 2022.docx [file pone.0263234.s008.docx]

***Editor’s comments***

| Editor comment | Authors response |
| --- | --- |
| Please review your reference list to ensure that it is complete and correct. If you have cited papers that have been retracted, please include the rationale for doing so in the manuscript text, or remove these references and replace them with relevant current references. Any changes to the reference list should be mentioned in the rebuttal letter that accompanies your revised manuscript. If you need to cite a retracted article, indicate the article’s retracted status in the References list and also include a citation and full reference for the retraction notice. | Please be informed that no articles were retracted nor were there any changes to the reference list of the manuscript. |
| My biggest concern is that the "views and perspectives" column in Table 3 is not clear. The phrases are too short and the table does not standalone. | Column renamed to “codes” to use the same jargon as Thomas and Harden. Phrases are short here because these were the codes used in the thematic synthesis process as per Thomas and Harden. The purpose of the table is to provide a bird's eye view of the synthesis and themes, each of these codes and themes are described in greater detail in the findings below (this is explained in the newly added line 218) |
| I am still concerned that describing coercion or massive power imbalances are described as good rapport (line 306) | Thank you for this possible interpretation of the selected quote, Bergenfeld et al’s interpretation of the quote was that the social position and knowledge of MHCPs was part of why mother trusted MHCPs and deferred vaccination decisions to the advice to the MHCP. This interpretation by the original author was made clearer by editing and moving the sentence “Bergenfeld et al. explained that….” (Line 301-303) |
| The very end indicates that there is a start date to the search strategy, but this is not included in the methods | This was clarified in the edits to line124-125 |
| -There may not be consensus about how to assess quality of qualitative papers, but there are methods, including eliminating ones not meeting a certain threshold | Quality assessment was described. CASP checklists were used and appended. There were no majorly flawed papers as described in the manuscript. The decision of the authors was to not exclude any qualitative papers as all papers contributed to the understanding of the perspectives. Please refer to line 158-159. |
| -I'd still like to see more differentiation between findings from many papers verses one or few papers, and perhaps a bit more clarity between HCP's words themselves and what is ascribed to them | Table 3 shows a clear picture of how many papers contributed to each finding. In the explanation of each paper there is clear indication of the number of papers that contributed to each finding as well from the citation and writing. A line explaining the table contents was added (Line 218).  All quotes included are directly from HCP participants in the respective papers. This was clarified in the following addition in line 218-219. |
| -One strength of this paper is the global nature; consider adding "global" to the title | This now has been amended to “**Views and experiences of maternal healthcare providers regarding influenza vaccine during pregnancy globally: a systematic review and qualitative evidence synthesis”** |
| -EMR should be spelled out in the abstract | This now has been amended |
| -line 113 - "looked for" is not very specific (perhaps identify and analyse?) | This now has been amended |
| -table 1: what about health care providers who are also pregnant? | This now has been amended |
| -line 190 should be "represented" | This now has been amended |
| -GP should be spelled out at first use | This now has been amended |
| -line 262 should be "including" | This now has been amended |
| -line 289: literacy is not the same as materials (literacy is a skill) | This now has been amended to “materials” |
| -line 357: should be "improved/strengthened" recordkeeping (record keeping should already be happening) | This now has been amended |
| -line 384: should be MHCP | This now has been amended |

***Reviewer’s comments***

| ***Reviewer comments*** | ***Authors responses*** |
| --- | --- |
| Title—Would consider “regarding” or “of” rather than “on” influenza vaccination | This now has been amended as” **Views and experiences of maternal healthcare providers regarding influenza vaccine during pregnancy globally: a systematic review and qualitative evidence synthesis”** |
| 308 to 311—these are different aspects of the conversation—the 1:1 nature and allowing women to ask questions, and then the use of passive language. Would separate into two sentences and discuss implications of each. Passive language sounds like it belongs in lines 314-318 re-emphasizing that is the woman’s choice | Thank you for this comment,  1-1 discussion was further explained with the inclusion of a quote to depict this. Please refer to line 306-313.  New paragraph was created to explain the use of passive language. A line was added to explain how the use of passive language proposes an option/choice for mothers (line 314-318) |
| 423-424 think more “attitudes” than “views” | This now has been amended, thank you |
| Think casting as barriers and facilitators is makes for much clearer delineation of the findings from the studies | Headings were added to delineate barriers and facilitators more clearly |
| 461 “trust and good rapport between MHCP and patient (or pregnant woman), clear national guidelines, and education and training for MHCPs | This now has been amended, thank you |
| 463-479 Seems like you need to discuss national guidelines in this section, since you included it among facilitators | National guideline was added to this section in line 490-492 |
| 485 would bold or italicize to mark this as a separate section | This now has been amended, thank you |
| 538 Would emphasize the importance of the influenza vaccine again here, protecting both mother and fetus. | This now has been amended as” Although influenza vaccine could prevent hospitalisation of pregnant women due to influenza infection, provide immunity to the new-born, increasing influenza vaccination uptake in pregnant women remains a challenge.” |
| Could also suggest there may be implications for uptake of other, newer vaccines | This now has been amended as” The suggestions developed in this review may be applicable to the uptake of other vaccines such as Covid-19 vaccine.” |
